# Soft Shear Sensing of Robotic Twisting Tasks Using Reduced-Order Conductivity Modeling

**DOI:** 10.3390/s25165159

**Published:** 2025-08-19

**Authors:** Dhruv Trehan, David Hardman, Fumiya Iida

**Affiliations:** Bio-Inspired Robotics Laboratory, University of Cambridge, Cambridge CB2 1PZ, UKfi224@cam.ac.uk (F.I.)

**Keywords:** soft sensors, robotic tool use, electrical impedance tomography

## Abstract

Much as the information generated by our fingertips is used for fine-scale grasping and manipulation, closed-loop dexterous robotic manipulation requires rich tactile information to be generated by artificial fingertip sensors. In particular, fingertip shear sensing dominates modalities such as twisting, dragging, and slipping, but there is limited research exploring soft shear predictions from an increasingly popular single-material tactile technology: electrical impedance tomography (EIT). Here, we focus on the twisting of a screwdriver as a representative shear-based task in which the signals generated by EIT hardware can be analyzed. Since EIT’s analytical reconstructions are based upon conductivity distributions, we propose and investigate five reduced-order models which relate shear-based screwdriver twisting to the conductivity maps of a robot’s single-material sensorized fingertips. We show how the physical basis of our reduced-order approach means that insights can be deduced from noisy signals during the twisting tasks, with respective torque and diameter correlations of 0.96 and 0.97 to our reduced-order parameters. Additionally, unlike traditional reconstruction techniques, all necessary FEM model signals can be precalculated with our approach, promising a route towards future high-speed closed-loop implementations.

## 1. Introduction

Soft stretchable sensors provide a way for robotic manipulators to monitor their external interactions, while still providing a compliant and flexible interface which can conform to objects being grasped [1]. As such, numerous stretchable sensing technologies have been developed for detecting external forces [2]. Many focus only on forces in the normal direction which, while useful, does not provide a complete picture [3]. The ability to detect additional shear forces provides information about tangential stimuli in situations including slip detection, texture recognition, and tool use [4,5]. Correspondingly, various soft shear sensors exist: Aksoy et al. demonstrate the use of capacitive strain sensors to measure shear forces up to a magnitude of 1N [6,7]; Platkiewicz et al. apply deformation-based shear sensing to a universal gripper to facilitate robust edge detection [8]; and vision-based tactile sensing methods, such as the biomimetic TacTip’s internal pin structure, allow shear forces to be recognized [9,10]. Still, most robotic shear sensing methods require layered materials or embedded components, limiting flexibility and increasing delamination risk. Examples include capacitive shear sensors which require multiple plates or electrodes [11,12], or approaches which rely on the stacking of small unit cells into larger lattice structures [13,14]. Optical fibers are one method for offloading the tactile sensory transducer away from the area of deformation [15,16], but they still rely on the embedding of an additional component: the fibers themselves. Domenici et al. noted how single-material solutions are promising candidates for tactile sensing [17], demonstrating shear sensing on a 1 mm wide strip of piezoelectric polymer.

Recently, tactile sensors based on electrical impedance tomography (EIT [18]) have been gaining popularity for soft robotic applications [19,20], using only a single conductive material as the basis of their bodies. By multiplexing between thousands of impedance measurements over a piezoresistive material, EIT-based sensors can simultaneously return vast amounts of rich data about different touches, materials [21], normal forces [22], proprioceptive shape [23], damages, and environmental conditions [24]. Their peripheral electrodes allow large-area sensors to be made from uninterrupted soft materials which are free to act as compliant skins [25]. Still, despite the importance of shear in dexterous manipulation tasks, few works have attempted to explicitly predict shear forces from EIT-based sensors’ output measurements. Park et al. [26] examined in simulation how anisotropic EIT (aEIT) could be used to generate tensor predictions, and Lee et al. [27] used aEIT with multiwall carbon nanotube compositions to enable directional displacement reconstructions over a soft hand-sized interface.

**Figure 1:**
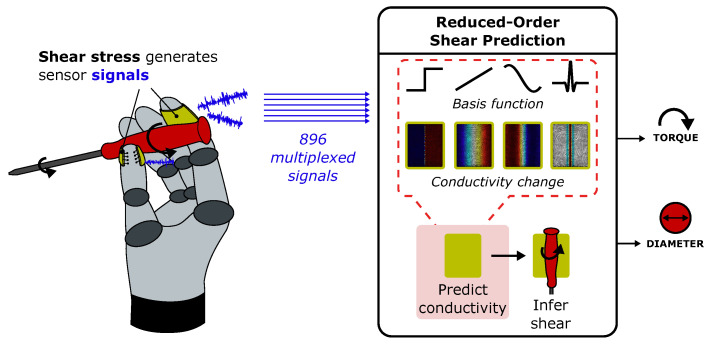
Reduced-order shear predictions using multiplexed impedance measurements. By using our reduced-order models of conductivity to interpret hundreds of incoming sensor signals, changes in torque and diameter can be straightforwardly monitored during robotic twisting tasks.

Here, we focus on how sensorized EIT fingertips can be used to monitor shear forces generated by tool use. The robotic use of rotational hand tools such as screwdrivers requires not only actuation [28], but also robust sensory feedback covering the large-area surfaces of the gripper [29,30]. Existing tactile EIT technologies are already capable of predicting the location and normal force of gripped objects such as screwdrivers [31]. Instead of predicting complex maps of conductivity to additionally monitor shear, we propose and develop a reduced-order approach which maps a fingertip’s 896 multiplexed measurements directly into a physically based reduced-order space, such that changes in a screwdriver’s torque and diameter can be intuitively tracked (Figure 1). In addition, by limiting our parameter space to two dimensions, a significant portion of the required FEM analysis can be precomputed, providing a path towards high-speed closed-loop implementations of tool use. Such a pipeline is a vital addition to robotic tool-use applications, where interpretation speed and efficiency are important considerations during complex manipulation tasks [32].

**Figure 2:**
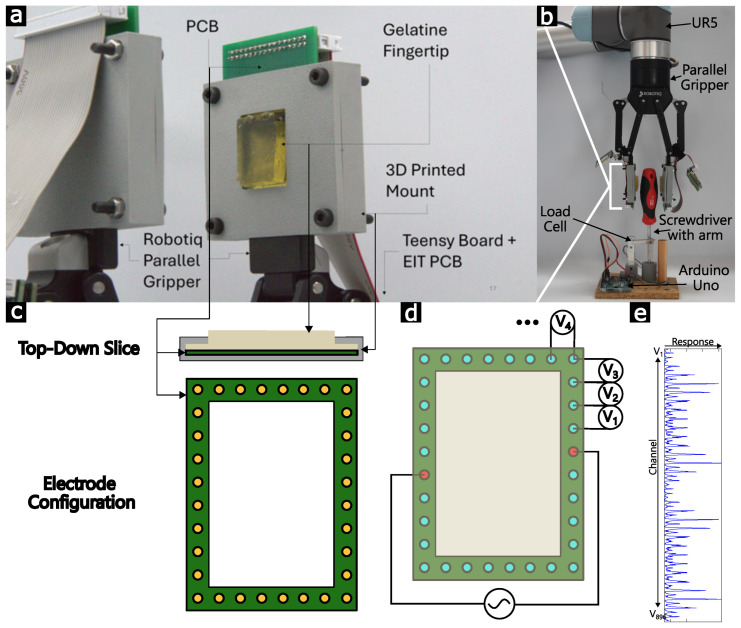
Experimental setup for shear prediction during twisting tasks. (**a**) A close-up of the sensor, consisting of a conductive hydrogel fingertip clamped to a 32-electrode PCB and a parallel gripper. (**b**) The full setup showing the parallel gripper attached to a UR5 robotic arm, used to grab and twist a screwdriver with a known length arm attached to a load cell connected to an Arduino Uno. (**c**) Slices of the sensor clamping the fingertip to the PCB and the electrode configuration of the PCB under the fingertip. (**d**) EIT diagram detailing how the opposite-adjacent methodology injects current into opposite electrodes and reads the voltage between all other adjacent pairs of electrodes. (**e**) An example measured frame from this setup, consisting of 896 multiplexed measurements.

Figure 2 shows the experimental setup through which we demonstrate our approach. A conductive hydrogel is used as a soft and compliant fingertip interface between the handle of a screwdriver and a two-finger gripper mounted to a robotic arm. The fingertip is surrounded by 32 electrodes, which take 896 multiplexed tetrapolar measurements per information frame. When the screwdriver is grasped and twisted, local shear and normal deformations of the hydrogel influence its conductivity, which in turn affects the measurements taken at the fringes, which we monitor with EIT equipment [33]. The EIT signals can then be compared to a precomputed signal bank for the given setup to determine the torque and diameter of the screwdriver used. The screwdriver is mounted in a torque-measuring rig to provide ground truth values during tool use. Further experimental details are given in Section 2.

In the following sections, we first show how the 896 measurements contain sufficient information to predict a screwdriver’s torque using a data-driven approach and hundreds of physical measurements. We then shift our focus to a more general and physically based approach, proposing five reduced-order models which relate twist-generated deformations to a fingertip’s conductivity. After selecting the best model, we demonstrate how this can be used to generate a precalculated signal bank from analytical FEM models, which we use to map changes in the screwdriver’s torque and diameter into an intuitive two-dimensional space. This proposed pipeline can be easily transferred to other sensor morphologies and tool uses, and we conclude by discussing how our approach could be beneficially integrated into increasingly dexterous robotic manipulation tasks.

## 2. Experimental Section

Actuation was performed by a two-finger parallel gripper (Robotiq 2F-140, Lévis, QC, Canada) serving as the end effector of a 6-DoF robotic arm (Universal Robots UR5, Odense, Denmark), both controlled using Python 3.12.4 via an attached PC.

Sensorized fingertips were joined to the inner surfaces of both gripper fingers, as seen in Figure 2. A gelatin-based hydrogel was used as the homogeneous soft sensorized surface, combining gelatin powder (240 bloom, MM ingredients, Wimborne, UK), water, glycerol (Fisher Scientific, Waltham, MA, USA), citric acid monohydrate (Fisher Scientific, Waltham, MA, USA), and salt (Sainsburys, London, UK) at a mass ratio of 1:1.5:1.5:0.2:0.1. The full gel preparation process is described by Hardman et al. in [34], where its stability is demonstrated over multiple months with changing environmental conditions, and in [24], where the material is evaluated for EIT applications. The gel was melted in an oven at 50 °C, cast into 3D-printed molds, and allowed to solidify overnight. Upon removal, the fingertip was simultaneously clamped onto the Robotiq gripper and onto a PCB introducing 32 peripheral electrodes, as shown in Figure 2. A ribbon cable joined each PCB to the boards used for the multiplexed measurements, namely Zhu et al.’s EIT-kit [33]. Throughout this work, the board was configured to measure tetrapolar AC impedance RMS values at 5 kHz using the traditional opposite-adjacent electrode configurations [18], returning 896 information channels in each frame, with a frame rate of 1.85 Hz. These measurements were serially transmitted to a connected PC where they were recorded and analyzed. Due to symmetry, only one of the fingertips is considered.

**Figure 3:**
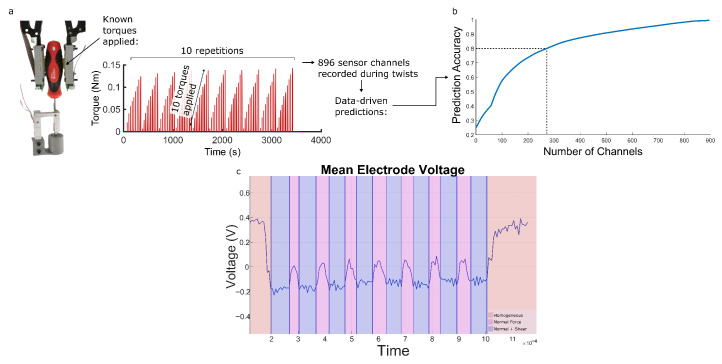
Collecting a torque dataset for a single screwdriver. (**a**) A total of 100 torque readings are taken over 1 h, and 896 responses are monitored. (**b**) Linear regression shows that the signals contain information about the torques, but its results cannot be generalized to new diameters. We instead propose a physically based approach that does not require new datasets for each new screwdriver. (**c**) A plot of the mean voltage across all EIT channels during no action, gripping a screwdriver, and twisting a gripped screwdriver.

**Figure 4:**
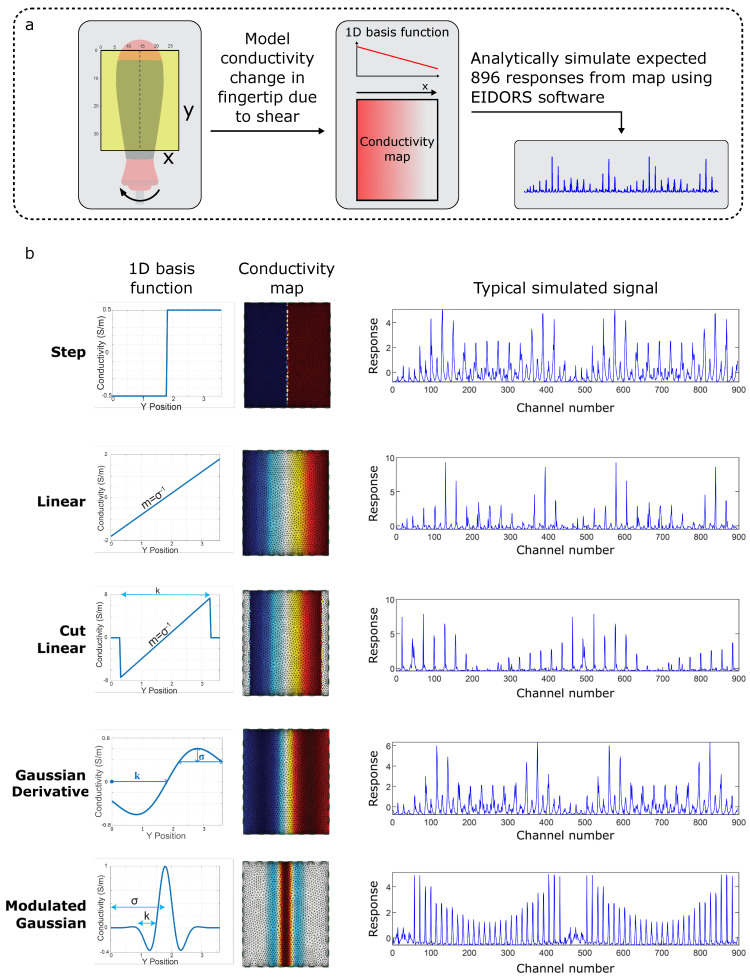
Details of the 5 proposed basis models. Five physically motivated 1D basis functions are introduced to model the shear. (**a**) The pipeline of generating signals using the proposed model. (**b**) A table of proposed model, EIDORS-simulated FEM of conductivity for that model and simulated signal calculated from the FEM.

**Figure 5:**
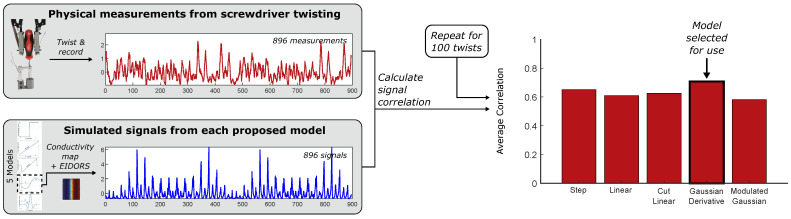
Correlation of the five models to ground truth measurements during basic twisting tasks. The Gaussian Derivative Model is selected for use throughout the rest of the paper.

**Figure 6:**
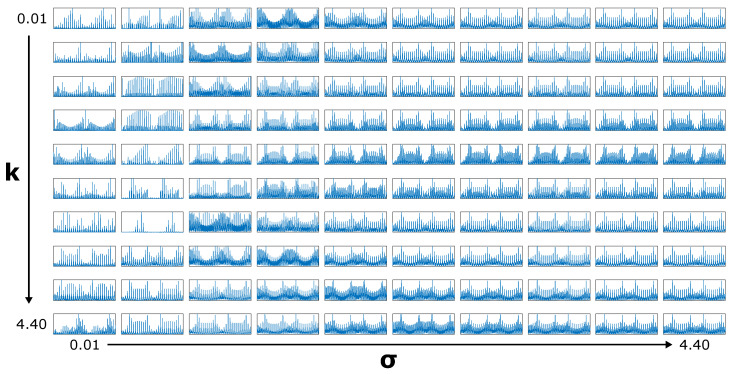
Precalculated signal bank generated by the 2-parameter Gaussian Derivative Model selected in Figure 5. For visualization purposes, a 10 × 10 grid is shown here: during calculations, the underlying grid is actually 100 × 100.

**Figure 7:**
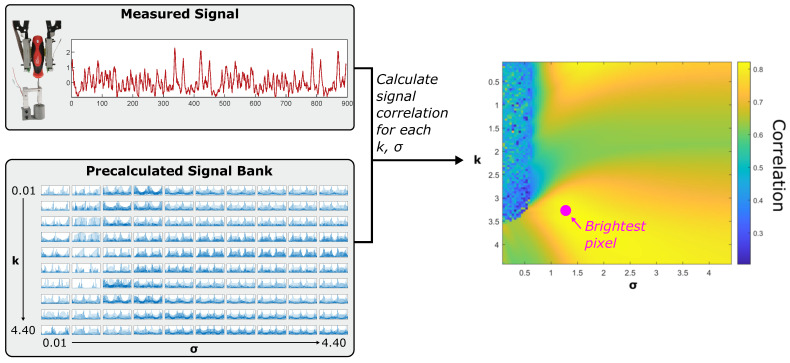
The generation of reduced-order correlation heatmaps using any measured signal and the precalculated signal bank.

Ground truth torque measurements were recorded using a custom rig: the shaft of a screwdriver was supported in a base using two bearings, allowing it to rotate freely. A 35 mm lever arm was rigidly attached to the shaft such that it contacted a calibrated load cell when the screwdriver was rotated: force measurements could be recorded at a known distance from the axis of rotation, such that the corresponding torques could be calculated. Measurements were read using an Arduino Uno connected to an HX711 amplifier and transmitted to a serially connected PC. In Figure 3, Figure 4, Figure 5, Figure 6 and Figure 7, recordings were taken from an actual screwdriver handle with TPU overmolding. This setup was used to record EIT readings for 10 different torques (Figure 3).

The EIT channels were ranked by calculating the correlation with the ground truth signal. Using a simple approach of using linear regression to give a weight to each channel, a reference accuracy for torque prediction was calculated for 896 channels. The relative accuracy to this reference was then calculated for the top N channels to produce Figure 3b. Using this dataset, we were first able to determine that for this specific case of twisting a screwdriver, the axis perpendicular to the screwdriver shaft was the most information-rich injection direction. Focusing on this dominant axis, five models were proposed.

The five models were compared against the data to determine the best model by calculating the cross correlation between the signal envelopes for the simulated and real data (subtracting the homogeneous signal). The Derivative of a Gaussian was discovered to give the best results and was used for the rest of the analysis (Figure 5).

The Derivative of a Gaussian was then used to pre-compute a 100 × 100 dataset of simulated signals, varying two characteristic parameters (k and σ) (Figure 6). This matrix can be used to create a heatmap of the cross correlation between each simulated signal and a measured data signal. Locating the global maxima of this heatmap gives the characteristic k and sigma for that measured signal.

**Figure 8:**
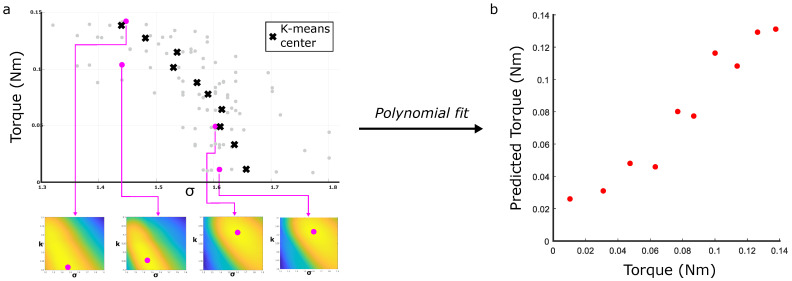
Predicting torques using the reduced-order model. (**a**) The σ value of the heatmaps’ brightest pixels for each of the torque measurements from Figure 3’s dataset, and the clustered central value for each torque. The heatmaps of 4 selected points (in pink) are visualized. (**b**) Predicting values of torque once a second-order polynomial is fitted to this data.

**Figure 9:**
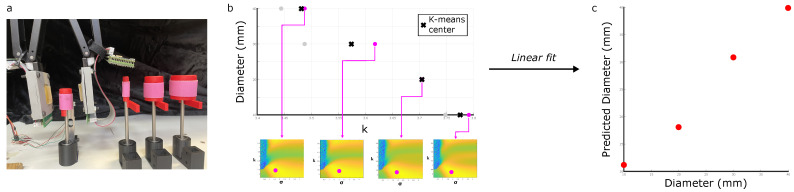
Predicting diameters using the reduced-order model. (**a**) Data was collected from four screwdriver diameters. (**b**) The k value of the heatmaps’ brightest pixels for the diameter measurements, and the clustered central value for each diameter. Note that some dots lie on top of each other. (**c**) Predicting values of diameter once a first-order polynomial is fitted to this data.

The characteristic parameters for each data point can be plotted against each other to observe the relationship between them and the physical parameters. By clustering the data (10 torques and 4 diameters), a relationship can be fit between the diameter and the k parameter, and between the torque and the σ parameter. This fit is used to calculate a predicted torque and predicted diameter as plotted in Figure 8 and Figure 9. This fit may also be applied to the raw un-clustered data.

In Figure 9, 3D-printed PLA cylinders were instead used, with constant diameters of 10, 20, 30, or 40 mm. To prevent slipping between the fingertip and cylinder at higher torques, thin silicone membranes were wrapped around the cylinders and fixed in place using Sil-Poxy adhesive. A torque was applied through rotation of the cylinders and held for 5 s. This was repeated 10 times for each diameter at a constant torque, from which some measurements were manually rejected due to obvious noise.

## 3. Results

Using Figure 2’s setup, we first collect a dataset of 100 responses when torques are applied to the screwdriver: 10 values, each repeated 10 times. For each, the responses of all 896 channels (Figure 2e) are monitored. The load cell’s ground truth during this data collection is shown in Figure 3. This torque dataset is first used to verify the feasibility of torque prediction from the fingertips, using linear regression (see Section 2). Figure 3 shows the mean voltage of the electrodes during an applied normal force and during a shear force with the same normal force used to determine a baseline value for the voltage before twisting occurs. Using 272 of the 896 channels achieves 80% accuracy (Figure 3), which increases in accuracy as more channels are used. This is a promising result, demonstrating that the fingertip’s signals contain sufficient information about the twist-based shears. However, this linear regression can only make such predictions for one screwdriver diameter: since its generation has not used any prior physical knowledge, new datasets (each lasting approximately 1 h) would have to be generated for each new screwdriver. We instead aim to predict the torque using physical insights into the conductivity deformations, such that our model can be used to monitor changes in both torque and diameter. We begin with the simplifying assumption, based on observations of the main deformation directions, that the conductivity only varies on the axis perpendicular to the axis of the torque (Figure 4a). We therefore focus our attention on modeling the 1D conductivity changes which are induced by a given torque and diameter.

Using this simplifying assumption, five physically based models are proposed in Figure 4, each consisting of a 1D basis function governed by up to two parameters (denoted σ and k for consistency). The Step Model (Equation (1)) assumes the hydrogel has constant compression/tension under torque. The Linear Model (Equation (2)) assumes linearly varying compression/tension. The Cut Linear Model (Equation (3)) expands on the Linear Model by considering the edge boundary effects. The Gaussian Derivative Model (Equation (4)) expands further on the Cut Linear Model and acts as a continuous, differentiable model. The Modulated Gaussian Model (Equation (5)) is inspired by the material bunching explored by [35]. For each model, EIDORS software (see Experimental Section) is used to model the set of 896 measurements which would be expected for the idealized conductivity map: Figure 4b shows how the behaviors of these signals vary for each of the five physically based models.

(1) $$\begin{array}{cc} \mathrm{Step}:\; & f\left(x,y\right)=\frac{1}{2}\left(y>k\right)-\frac{1}{2}\left(y\leq k\right) \end{array}$$

(2) $$\begin{array}{cc} \mathrm{Linear}:\; & f\left(x,y\right)=\frac{y-k}{\sigma } \end{array}$$

(3) $$\begin{array}{cc} \mathrm{Cut}\;\mathrm{Linear}:\; & f\left(x,y\right)=\frac{y-c}{\sigma }\;\mathrm{for}\;c-\frac{k}{2}\geq y\leq c-\frac{k}{2} \end{array}$$

(4) $$\begin{array}{cc} \mathrm{Gaussian}\;\mathrm{Derivative}:\; & f\left(x,y\right)=-\frac{\left(y-k\right)}{\sigma^{2}}e^{-\frac{{\left(y-k\right)}^{2}}{2\sigma^{2}}} \end{array}$$

(5) $$\begin{array}{cc} \mathrm{Modulated}\;\mathrm{Gaussian}:\; & f\left(x,y\right)=e^{-\frac{{\left(y-c\right)}^{2}}{2\sigma^{2}}}\cos\left(k(y-c)\right) \end{array}$$

To select the model which best represents the physical effects of our twisting tasks, the simulated signal for each model is compared to Figure 3’s measured signals, and the average cross correlation is calculated in order to rank the models. Using this ranking, the Gaussian Derivative Model is found to be the best selection for approximating the conductivity over the surface (Figure 5), with the highest cross correlation value. Henceforth, the Gaussian Derivative Model is used to generate all simulated signals unless otherwise specified.

The shape of the Gaussian Derivative Model’s conductivity map is governed by two parameters: k and σ. Varying these varies not only the shape of the map but also the simulated signals. These signals can be precomputed before any analysis takes place, reducing real-time computation: a 100 × 100 × 896 (k × σ × channels) signal bank of simulated measurements is generated using the model. The range of values used for both is 0.01–4.4 (4.4 being the maximum *y* value). For visualization purposes, Figure 6 shows a reduced 10 × 10 × 896 signal bank, where the effect of the varying parameters on the signal shapes is clearly visible.

Using the precomputed matrix, a unique heatmap can then be calculated for any recorded measurement by calculating its cross correlation with each signal in the precomputed matrix (Figure 7). This heatmap visualizes the effect of the model’s two parameters on the simulated signal accuracy, and it can be used to directly compare the physical effects of changing torques and diameters. Darker areas of low value indicate a mismatch between the simulations and physical measurements, while lighter yellow areas suggest parameter ranges in which the conductivity maps lead to accurate signals. The global maxima (i.e., the heatmap’s brightest pixel) defines a characteristic pair of parameters for each measured signal; we next examine how these abstracted visualizations lead to physically based insights into varying torques and diameters during our twisting tasks.

To explore how variations in torque affect this mapping, the dataset shown in Figure 3 is used to generate 99 characteristic heatmaps, from which σ values of their brightest pixels are plotted in grey in Figure 8a. Four representative heatmaps are shown, with their σ values marked by the pink dots. Given the variance in the ground truth torque values which were recorded, K-means clustering was used to sort the torques and characteristic parameters into groups: the black crosses mark their centers, with a correlation of −0.9588 between torque and σ (compared to −0.6552 before clustering occurred). Once a second-order polynomial is fitted to these points (see Section 2), Figure 8 shows the corresponding torque predictions of the clustered data.

Similarly, the brightest pixel’s k value can be used as an indicator of the screwdriver’s diameter: Figure 9a shows the set of four cylinders (with diameters 10, 20, 30, and 40 mm) from which data was collected. Figure 9 plots the variation in diameter with *k*: though less data was available, it is noted that many of these points lie on top of each other. The diameter and k axes have a correlation of −0.9746; once a first-order polynomial is used to map these to predictions, Figure 9 shows a clear linear fit.

## 4. Conclusions

In this work, we have explored the application of five physically based reduced-order models for soft shear sensing using electrical impedance tomography (EIT), focusing on the twisting of a screwdriver. Though we have focused specifically on directly predicting tool-use metrics, we expect our sensors to respond to and be able to predict more general shear forces. EIT’s single-material implementation maintains a soft and flexible interface at the tactile sensing area, requiring no internally embedded gauges or electrodes. A Gaussian Derivative Model was selected as the model which best represented EIT’s physical changes in conductivity, and was coupled with EIDORS simulations to precalculate a signal bank which could be directly compared to physical measurements coming from the fingertips to determine the characteristic parameters for the task. The two parameters which govern the reduced-order model—σ and *k*—were found to correlate with physical screwdriver torques and diameters, with respective correlation magnitudes of 0.96 and 0.97. The model correlates with experimental data for k > 3.5∪σ > 0.6. Our pipeline enables the direct prediction of task-based parameters, with FEM calculations being offloaded to precalculations to increase the speed with which our approach can be implemented. Future work will aim to incorporate the prediction of more twisting task-based parameters, including 3D forces, localizations, and screw identification, as well as the decoupling of simultaneous normal and shear forces. Further investigation into the ranges of diameter and torque at which the link between the simulated model and reality holds can also be undertaken. Further bridging the gap between the simulated data and experimental data would allow for more robust prediction and open up the possibility for implementing control through simulation [36]. Though we consider only screwdriver grasping in this work, we anticipate that our physically based approach and signal-bank pipeline could be adapted to a range of soft robotic tool-use tasks, providing a route towards application-driven high-speed sensor-driven dexterity.

## Data Availability

Data is available upon reasonable request.
